# Healthcare providers’ knowledge and clinical practice surrounding shigellosis — DocStyles Survey, 2020

**DOI:** 10.1186/s12875-023-02213-3

**Published:** 2023-12-13

**Authors:** Julia C. Haston, Laura Ford, Kayla L. Vanden Esschert, Ian D. Plumb, Naeemah Logan, Louise K. Francois Watkins, Amanda G. Garcia-Williams

**Affiliations:** 1 Epidemic Intelligence Service Program, Centers for Disease Control and Prevention, Atlanta, GA USA; 2 Division of Foodborne, Waterborne, and Environmental Diseases, Centers for Disease Control and Prevention, Atlanta, GA USA

**Keywords:** *Shigella*, Transmission, Health care provider education

## Abstract

**Background:**

Shigellosis is an acute diarrheal disease transmitted through contaminated food, water, objects, poor hand hygiene, or sexual activity. Healthcare providers (HCP) may not be aware of the multiple routes of *Shigella* transmission, populations at increased risk, or importance of antibiotic susceptibility testing (AST). This study assessed HCP knowledge and clinical practices regarding shigellosis and antibiotic resistance.

**Methods:**

Porter Novelli Public Services administered a web-based survey (Fall DocStyles 2020) to HCP in the United States. Pediatricians, primary care physicians, nurse practitioners, and physician assistants completed questions about knowledge and clinical practice of acute diarrhea and shigellosis.

**Results:**

Of 2196 HCP contacted, 1503 responded (68% response rate). Most identified contaminated food (85%) and water (79%) as routes of *Shigella* transmission; fewer recognized person-to-person contact (40%) and sexual activity (18%). Men who have sex with men (MSM) were identified as being at risk for shigellosis by 35% of respondents. Most reported counseling patients to wash hands (86%) and avoid food preparation (77%) when ill with shigellosis; 29% reported recommending avoiding sex. Many HCP reported treating shigellosis empirically with ciprofloxacin (62%) and azithromycin (32%), and 29% reported using AST to guide treatment.

**Conclusions:**

We identified several gaps in shigellosis knowledge among HCP including MSM as a risk group, person-to-person transmission, and appropriate antibiotic use. Improving HCP education could prevent the spread of shigellosis, including drug-resistant infections, among vulnerable populations.

**Supplementary Information:**

The online version contains supplementary material available at 10.1186/s12875-023-02213-3.

## Background

Shigellosis, an enteric disease caused by bacteria of the *Shigella* genus, infects approximately 450,000 people annually in the United States [1, 2]. Although *Shigella* may infect any human host, children 1–10 years of age have the highest rates of culture-confirmed *Shigella* infection in the United States, and between 2009 and 2018, 54.5% of shigellosis outbreaks occurred in childcare facilities [3, 4]. Other populations at increased risk include persons experiencing poverty or homelessness, men who have sex with men (MSM), refugees, and travelers to low resource countries [1, 5, 6]. *Shigella* can spread through person-to-person contact, food, water, fomites, and sexual contact, and it is one of the most contagious enteric pathogens, requiring as few as ten organisms to cause an infection [7]. Unlike many other enteric bacteria, over 80% of *Shigella* outbreaks reported to the National Outbreak Reporting System (NORS) between 2009 and 2018 were thought to occur through person-to-person contact, and sporadic cases of *Shigella* are most commonly attributed to person-to-person contact, international travel, and foodborne transmission [3, 6]. Waterborne transmission is a less common route of transmission in the United States, but 4%–16% of sporadic *Shigella* cases in the United States may be water-related [2, 6]. Direct and indirect sexual contact is another way shigellosis can spread, although it is not clear how common this mode of transmission is [8, 9].

Most people with symptomatic *Shigella* infection experience a gastrointestinal illness with diarrhea, abdominal pain, and occasionally fever, but severe complications can also occur [10]. Testing the stool of patients with shigellosis-like symptoms by culture or by culture-independent diagnostic test (and if positive, confirming with culture) is recommended [1]. Although most cases of shigellosis resolve with supportive care, empiric antibiotics have been shown to shorten the duration of illness and reduce shedding in the stool, and are recommended for immunocompromised patients, patients < 3 months of age, international travelers, and patients with severe symptoms [10–12]. If antibiotics are needed, antibiotic susceptibility testing (AST) should be conducted, and selection of antibiotics should be tailored to the susceptibility profile of the *Shigella* isolate. All patients with shigellosis should receive guidance to avoid swimming, food preparation, and sexual activity while ill and practice frequent handwashing [13].

Antimicrobial resistance is increasing to antibiotics used to treat shigellosis, including fluoroquinolones and macrolides, and drug-resistant *Shigella* is now considered a serious threat to human health by the Centers for Disease Control and Prevention (CDC) [14]. CDC surveillance data from 2021 indicate that of all *Shigella* isolates tested with AST, 28% were resistant to ciprofloxacin and 44% were resistant to azithromycin [15]. Drug-resistant *Shigella* have been implicated in multiple outbreaks and have been frequently reported among MSM [16–19]. Inappropriate antibiotic use can lead to both treatment failure and selection for antibiotic resistance [1, 20].

Healthcare providers (HCP) are uniquely positioned to take an active part in controlling the spread of *Shigella* in their communities. Previous studies have found gaps in HCP knowledge of *E. coli* and other foodborne bacterial pathogens [21–23]. The objectives of this study were to identify gaps in HCP knowledge of shigellosis epidemiology and characterize management practices to determine how HCP might improve clinical management and education of patients with shigellosis.

## Methods

### Study design and sample selection

DocStyles is a web-based survey developed by Porter Novelli Public Services and administered twice yearly to samples of HCP that includes questions about providers’ knowledge of several health issues [24]. The panel was constructed to prioritize primary care physicians (defined as family practitioners and internists) and also included obstetricians/gynecologists, pediatricians, nurse practitioners, and physician assistants. The survey used in this study was conducted from September 14–October 26, 2020, by SERMO, a global market research company, and included 135 questions. Participants were clinicians in the United States who have been in practice for at least three years, and eligibility was verified by telephone confirmation at their place of work. Participation was optional and respondents were paid up to $72 for their participation. A total of 2196 HCP were invited to answer the questions regarding *Shigella* and acute diarrhea. Obstetricians/gynecologists were not included in this section of the survey due to scope of practice.

CDC licenses access to results of the DocStyles surveys post-collection from Porter Novelli. Porter Novelli is not subject to CDC institutional review board review; however, they adhere to all professional standards and codes for market research. Survey respondents were informed that their answers were being used for market research and they could exit the survey at any time. No personal identifiers were included in the data file provided to CDC. This secondary analysis was reviewed by CDC in accordance with CDC policy, pursuant to applicable US regulations.

### Survey design

The DocStyles survey contained 15 questions about acute diarrhea and *Shigella*, including questions about knowledge and perceptions of *Shigella* transmission and prevention, as well as clinical practice regarding diagnostic testing and treatment (Supplementary Table S1). Respondents were encouraged to select all applicable answer choices for each question, and the order of choices was randomized for each participant. In addition to survey questions about knowledge and treatment of specific health issues, demographic and occupational data were collected, including gender, age, work setting, years in practice, geographic region, community setting, number of patients seen per week, and whether the provider treats pediatric patients.

The outcomes evaluated were percentages of participants who selected each response option, and the percentages of correct answers for the knowledge-based questions (questions 1–3). For questions 1–3, a correct answer was considered as any answer except “none of the above”. Answer choices were analyzed overall, by demographic characteristics, and by provider specialty. Analysis of questions meant to characterize behavior regarding HCP diagnosis and treatment practices among patients with acute diarrhea (questions 8–11) were restricted to providers who reported seeing at least one patient per week with acute diarrhea (*N* = 1287). Likewise, analysis of questions meant to characterize behavior regarding HCP treatment of patients with shigellosis (questions 13–15) were restricted to providers who reported seeing at least one patient per month with laboratory-confirmed *Shigella* infection (*N* = 391).

### Statistical analysis

The cohort was described overall and by provider specialty. Frequency of answer choices by specialty were compared using Chi square difference of proportions. Fisher exact tests were used for comparisons in which ≥ 25% of cells were expected to have counts of < 5. Statistical significance was determined at *p*-values of < 0.05. All analyses were performed using SAS (version 9.4; Cary, NC).

## Results

### Description of sample

Of 2196 HCP who were eligible to answer questions about acute diarrhea and shigellosis, 1503 HCP completed the survey (68% response rate), primarily consisting of family practitioners and internists (67%, Table 1). The majority of respondents were men (60%), and the median age was 45 years (range 25–89). Most of the respondents worked in group outpatient practices (70%); 14% practiced inpatient medicine. The sample was geographically dispersed across the United States and represented primarily urban or suburban populations. The median duration in practice was 15 years (range 3–48), and the median number of patients seen per week was 100 (range 10–500). Most providers (86%) reported treating patients with acute diarrhea on a weekly basis, and 26% reported diagnosing at least one case of shigellosis monthly.

**Table 1:** Demographics and clinical practice characteristics of survey respondents, overall and by provider specialty

	**Total** **N (%)**	**Provider Specialty** **N (%)**				
		**Family Practitioner**	**Internist**	**Pediatrician**	**Nurse Practitioner**	**Physician Assistant**
**Total**	1503 (100.0)	441 (29.3)	559 (37.2)	252 (16.8)	134 (8.9)	117 (7.8)
**Gender**						
Male	905 (60.2)	300 (68.0)	399 (71.4)	143 (56.7)	24 (17.9)	39 (33.3)
Female	598 (39.8)	141 (32.0)	160 (28.6)	109 (43.3)	110 (82.1)	78 (66.7)
**Age**						
25–34 years	216 (14.4)	50 (11.3)	67 (12.0)	22 (8.7)	28 (20.9)	49 (41.9)
35–44 years	471 (31.3)	124 (28.1)	190 (34.0)	66 (26.2)	49 (36.6)	42 (35.9)
45–54 years	438 (29.1)	144 (32.7)	166 (29.7)	81 (32.1)	29 (21.6)	18 (15.4)
55–64 years	282 (18.8)	95 (21.5)	100 (17.9)	62 (24.6)	21 (16.7)	4 (3.4)
> 65 years	96 (6.4)	28 (6.3)	36 (6.4)	21 (8.3)	7 (5.2)	4 (3.4)
**Region**						
Midwest	338 (22.5)	88 (20.0)	133 (23.8)	64 (25.4)	31 (23.1)	22 (18.8)
South	329 (21.9)	117 (26.5)	108 (19.3)	47 (18.7)	35 (26.1)	22 (18.8)
Northeast	513 (34.1)	142 (32.2)	180 (32.2)	95 (37.7)	44 (32.8)	52 (44.4)
West	323 (21.5)	94 (21.3)	138 (24.7)	46 (18.3)	24 (17.9)	21 (17.9)
**Community Setting**						
Urban	543 (36.1)	114 (25.9)	249 (44.5)	96 (38.1)	45 (33.6)	39 (33.3)
Suburban	777 (51.7)	250 (56.7)	272 (48.7)	131 (52.0)	63 (47.0)	61 (52.1)
Rural	183 (12.2)	7 (17.5)	38 (6.8)	25 (9.9)	26 (19.4)	17 (14.5)
**Work Setting**						
Individual Outpatient practice	247 (16.4)	79 (17.9)	89 (15.9)	19 (7.5)	29 (21.6)	31 (26.5)
Group Outpatient practice	1047 (69.7)	342 (77.6)	343 (61.4)	208 (82.5)	85 (63.4)	69 (59.0)
Inpatient Practice	209 (13.9)	20 (4.5)	127 (22.7)	25 (9.9)	20 (14.9)	17 (14.5)
**Years in Practice**						
< 10 years	435 (28.9)	105 (23.8)	161 (28.8)	52 (20.6)	43 (32.1)	74 (63.2)
10–19 years	551 (36.7)	169 (38.3)	216 (38.6)	82 (32.5)	56 (41.8)	28 (23.9)
20–29 years	367 (24.4)	126 (28.6)	127 (22.7)	77 (30.6)	26 (19.4)	11 (9.4)
> 30 years	150 (10.0)	41 (9.3)	55 (9.8)	41 (16.3)	9 (6.7)	4 (3.4)
**Patients Seen per Week**						
< 50	129 (8.6)	22 (5.0)	50 (8.9)	18 (7.1)	31 (23.1)	8 (6.8)
50–99	580 (38.6)	141 (32.0)	230 (41.1)	103 (40.9)	57 (42.5)	49 (41.9)
100–149	561 (37.3)	194 (44.0)	192 (34.3)	95 (37.7)	39 (29.1)	41 (35.0)
> 150	233 (15.5)	84 (19.0)	87 (15.6)	36 (14.3)	7 (5.2)	19 (16.2)
**Pediatric Patients Seen**						
Yes	1065 (70.9)	400 (90.7)	232 (41.5)	252 (100)	90 (67.2)	91 (77.8)
No	438 (29.1)	41 (9.3)	327 (58.5)	0 (0)	44 (32.8)	26 (22.2)
**Average Number of Patients Seen per Week with Acute Diarrhea** **Mean (SD)**	7.0 (12.1)	6.9 (16.0)	6.8 (9.9)	7.8 (7.7)	6.3 (12.0)	7.2 (12.8)
**Average Number of Patients Seen per Month with Positive *Shigella* Test** **Mean (SD)**	1.3 (7.8)	1.3 (7.9)	1.8 (10.5)	0.7 (1.7)	0.4 (1.6)	0.7 (2.5)

### Knowledge of shigellosis transmission and prevention

Respondents correctly identified possible routes of *Shigella* transmission as contaminated food (85%), contaminated water (79%), fomite transmission (33%), person-to-person contact (44%), and sexual activity (18%) (Table 2). Many providers identified international travelers (80%) and people living in poverty or experiencing homelessness (79% and 70%, respectively) as being at risk for shigellosis, and some correctly selected other at-risk populations including children (57%) and MSM (35%). HCP reported encouraging patients with shigellosis to wash hands frequently (95%) and avoid food preparation (85%), while avoiding swimming and sex were less frequently encouraged. In total, 7% of providers chose all correct responses for routes of transmission, 21% correctly identified all populations at risk, and 24% encouraged all recommended prevention strategies (Supplementary Table S2).

**Table 2:** Healthcare provider responses to *Shigella* knowledge-based questions, overall and by provider specialty

	**Total** **N (%)**	**Provider Specialty** **N (%)**					**Chi-square *p*-value**
	**1503 (100.0)**	**Family Practitioner** **441 (29.3)**	**Internist** **559 (37.2)**	**Pediatrician** **252 (16.8)**	**Nurse Practitioner** **134 (8.9)**	**Physician Assistant** **117 (7.8)**	
**What are some ways adults get infected with *Shigella*? *Select all that apply***							
Contaminated food	1276 (84.9)	378 (85.7)	471 (84.3)	223 (88.5)	110 (82.1)	94 (80.3)	0.23
Contaminated water	1185 (78.8)	357 (81.0)	425 (76.0)	190 (75.4)	113 (84.3)	100 (85.5)	**0.03**
Touching fomites	489 (32.5)	141 (32.0)	172 (30.8)	97 (38.5)	46 (34.3)	22 (28.2)	0.19
Person-to-person contact	593 (39.5)	167 (37.9)	208 (37.2)	126 (50.0)	52 (38.8)	40 (34.2)	** < 0.01**
During international travel	837 (55.7)	258 (58.5)	311 (55.6)	150 (59.2)	70 (52.2)	48 (41.0)	** < 0.01**
Sexual activity	264 (17.6)	75 (17.0)	96 (17.2)	44 (17.5)	30 (22.4)	19 (16.2)	0.65
None of these	17 (1.1)	3 (0.7)	8 (1.4)	0 (0)	5 (3.7)	1 (0.9)	**0.02**^**a**^
**Who is at risk for *Shigella* infection? *Select all that apply***							
Children	850 (56.6)	235 (53.3)	293 (52.4)	197 (78.2)	68 (50.7)	57 (48.7)	** < 0.01**
Refugees	1013 (67.4)	318 (72.1)	362 (64.8)	176 (69.8)	86 (64.2)	71 (60.7)	**0.04**
International travelers	1205 (80.2)	363 (82.3)	440 (78.7)	202 (80.2)	105 (78.4)	95 (81.2)	0.67
People living in poverty	1194 (79.4)	360 (81.6)	440 (78.7)	198 (78.6)	105 (78.4)	91 (77.8)	0.76
Men who have sex with men	519 (34.5)	148 (33.6)	225 (40.3)	72 (28.6)	42 (31.3)	32 (27.4)	** < 0.01**
People experiencing homelessness	1050 (69.9)	317 (71.9)	377 (67.4)	179 (71.0)	92 (68.7)	85 (72.6)	0.54
None of these	26 (1.7)	6 (1.4)	13 (2.3)	0 (0)	6 (4.5)	1 (0.9)	**0.01**^**a**^
**Which behaviors do you encourage adult patients (18 +) who are actively sick with *Shigella* infection to follow?**^**b**^ ***Select all that apply***							
Avoid swimming	673 (49.3)	185 (44.5)	253 (48.1)	121 (58.2)	63 (55.8)	51 (49.5)	**0.01**
Avoid preparing food for others	1160 (84.9)	352 (84.6)	441 (83.8)	183 (88.0)	93 (82.3)	91 (88.4)	0.47
Wash hands frequently	1291 (94.5)	397 (95.4)	491 (93.4)	195 (93.8)	108 (95.6)	100 (97.1)	0.43
Avoid sex	442 (32.4)	121 (29.1)	167 (31.8)	68 (32.7)	48 (42.5)	38 (36.9)	0.08
None of these	13 (1.0)	5 (1.2)	7 (1.3)	1 (0.5)	0 (0)	0 (0)	0.46^a^

Boldface *p*-value indicates statistical significance (*p* < 0.05)

^a^Fisher’s exact test used rather than Chi square

^b^Excludes 137 respondents who selected “Question not applicable to me” (*N* = 1366)

Sexual activity was the least frequently selected route of transmission (18%) and MSM were the least frequently identified group at-risk for shigellosis (35%). Approximately one third of providers endorsed encouraging their adult patients with shigellosis to avoid sex. Among 1,042 providers who see adult patients with acute diarrhea at least once weekly, 22% report asking about sexual practices upon the initial consult, and 10% provide sexual health education. The most common barrier to discussing shigellosis with patients was that shigellosis is often diagnosed after the patient encounter has ended (32%). Less frequently perceived barriers included lack of time (17%) and lack of access to educational resources (17%).

Shigellosis knowledge varied by provider specialty; more pediatricians identified children as an at-risk population compared with others (78% compared with 49–57%), while more internists correctly identified MSM as an at-risk population (40% compared with 27–35%). In addition to provider specialty, age was found to be associated with shigellosis knowledge. More HCP aged 45–64 years chose all correct answers for knowledge-based questions compared with providers in younger or older age groups.

### Diagnosis and treatment of *Shigella* infections

Among 1287 HCP who reported seeing patients each week with acute diarrhea, 29% reported routinely using a culture-independent diagnostic test and 64% reported routinely requesting a stool culture, 20% of whom reported routinely requesting AST (Table 3). Eighteen percent of respondents reported routinely prescribing antibiotics in patients with acute diarrhea.

**Table 3:** Diagnostic and treatment actions reported by healthcare providers of patients with acute diarrhea or shigellosis

	**Responses among HCP who regularly treat patients with acute diarrhea or shigellosis** **N (%)**		**Chi-square *p*-value**
	**Acute diarrhea**^**a**^ **1287 (85.6)**	**Shigellosis**^**b**^ **391 (26.0)**	
**When you provide a consult for a patient with acute diarrhea, do you routinely: *Select all that apply***			
Ask about sexual practices^c^	239 (18.6)	111 (28.4)	** < 0.01**
Provide sexual health education^c^	112 (8.7)	67 (17.1)	** < 0.01**
Use a culture-independent diagnostic test	375 (29.1)	166 (42.5)	** < 0.01**
Request a stool culture	826 (64.2)	270 (69.1)	0.08
Request antibiotic susceptibility testing^d^	166 (20.1)	90 (33.3)	** < 0.01**
Treat with antibiotics	234 (18.2)	116 (29.7)	** < 0.01**
None of these	315 (24.5)	46 (11.8)	** < 0.01**
**To whom do you usually prescribe antibiotics?**^**e**^ ***Select all that apply***			
International travelers	602 (46.8)	202 (51.7)	0.09
Hospitalized patients	426 (33.1)	218 (55.8)	** < 0.01**
Men who have sex with men	185 (14.5)	116 (29.7)	** < 0.01**
Adults	232 (18.0)	184 (47.1)	** < 0.01**
Children	127 (9.9)	150 (38.7)	** < 0.01**
None of these	391 (30.4)	21 (5.4)	** < 0.01**

Abbreviations: HCP, healthcare providers

Boldface indicates statistical significance (*p* < 0.05)

^a^HCP who report treating patients with acute diarrhea at least once per week (*N* = 1297)

^b^HCP who report treating patients with positive *Shigella* tests at least once per month (*N* = 391)

^c^Includes pediatricians

^d^Among those who routinely request a stool culture

^e^For the analysis of HCP who regularly treat patients with acute diarrhea, this question was phrased, “Which of the following types of patients with acute diarrhea do you usually treat empirically with antibiotics before a pathogen is identified?”. For the analysis of HCP who regularly treat patients with positive *Shigella* tests, this question was phrased, “Among patients with a positive test for *Shigella,* to whom do you usually prescribe antibiotics?”

Among 391 providers who regularly treat patients with confirmed shigellosis, 52% usually prescribe antibiotics to returned international travelers with shigellosis; similarly, 47% of providers who regularly treat patients with acute diarrhea prescribe empiric antibiotics to international travelers with acute diarrhea. A higher proportion of HCP report prescribing antibiotics for children (38% vs 10%), MSM (30% vs 14%), hospitalized patients (56% vs 33%), or other adults (47% vs 18%) diagnosed with *Shigella* compared with HCP treating patients presenting with acute diarrhea. 

**Fig. 1:**
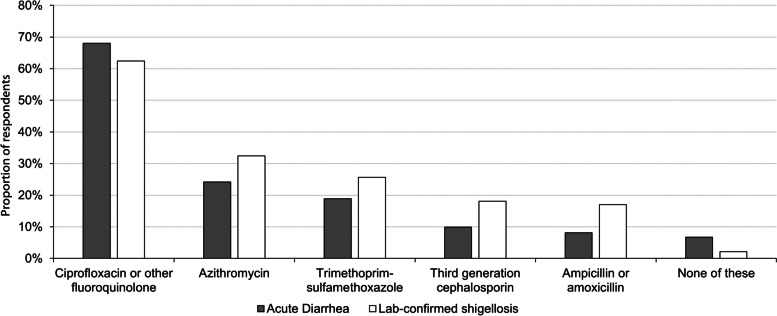
Antibiotics reported to be prescribed for patients with acute diarrhea or shigellosis among healthcare providers. “Acute Diarrhea” category represents only respondents who report seeing at least one patient per week with acute diarrhea and usually prescribe antibiotics (*N* = 896). “Positive *Shigella* test” category represents only respondents who report seeing at least one patient with *Shigella* each month and usually prescribe antibiotics (*N* = 370)

For the treatment of confirmed *Shigella* infection, the most commonly reported antibiotics were fluoroquinolones (Fig. 1). Among 370 HCP who regularly see patients with shigellosis and usually prescribe antibiotics, 62% reported commonly choosing ciprofloxacin or another fluoroquinolone, 32% reported commonly choosing azithromycin, and 26% reported prescribing trimethoprim-sulfamethoxazole. Among HCP regularly seeing patients with shigellosis and choosing to treat with antibiotics, 29% reported routinely ordering AST for patients with acute diarrhea.

### Sources of information

To inform antibiotic treatment, HCP who routinely treat acute diarrhea most frequently reported using recommendations and information from the Centers for Disease Control and Prevention (CDC) (67%), followed by scientific articles, textbooks, or professional organizations (45%), and AST results (39%) (Supplementary Figure S1).

## Discussion

In this cross-sectional survey of a sample of U.S. healthcare providers, we identified gaps in knowledge of shigellosis transmission, risk factors, prevention strategies, and diagnostic and treatment practices. Many HCPs reported empirically prescribing antibiotics to patients with acute diarrhea without routinely ordering stool testing, as is recommended by the Infectious Diseases Society of America clinical practice guidelines for infectious diarrhea [10]. Without the information provided by AST, providers may not be choosing the most appropriate medications for their patients. Public health practitioners can support HCP knowledge of shigellosis by providing accurate and up-to-date information, particularly through the sources reportedly used most by providers, including the CDC as well as scientific articles, textbooks, and professional organizations.

There is a gap in knowledge of shigellosis transmission and risk factors. In the United States, most sporadic cases and outbreaks of shigellosis are attributed to person-to-person contact; foodborne and waterborne transmission is less commonly implicated [3, 6, 20]. Although most providers identified contaminated food and water as potential modes of *Shigella* transmission, fewer than 40% identified other modes of transmission, including fomites, person-to-person contact, or sexual activity. Fewer than one-fourth of HCP reported asking adult patients with diarrhea about sexual practices and even fewer reported providing guidance to prevent sexual transmission. Addressing these knowledge gaps could have a large public health impact by improving timely diagnosis and reducing transmission.

MSM are at increased risk of shigellosis [6]; in particular, several studies have shown an increase in ciprofloxacin-resistant, azithromycin-resistant, or extensively drug-resistant infections among MSM [25–27]. The increase in drug-resistant infections is concerning for HIV-infected MSM who may have weakened immune systems and can be disproportionately affected by shigellosis [28]. In our study, 35% of HCP identified MSM as a population at increased risk of shigellosis. HCP have a critical role to play in reducing health disparities, including those related to race, ethnicity, income, and sexual behavior, which are known to exist with shigellosis [4, 5, 29–31]. Health disparity education for HCP is often inadequate, poorly funded, not universal, and not standardized [32, 33]. Therefore, it is imperative that clinical training, such as formal education in medical school, residency training for physicians in primary care, or courses in continuing medical education, addresses social determinants that can increase risk of enteric pathogens among patients, such as those described in the survey. Initial efforts could include providing toolkits and active health education to HCP who routinely treat children, refugees, people living in poverty or experiencing homelessness, travelers, or MSM.

Our study also suggests that HCP may not consider shigellosis when patients present with acute diarrhea. Although shigellosis is less common than some other causes of acute diarrhea in the United States [34], it is still important for HCP to appropriately assess shigellosis risk factors in their patients with acute diarrhea and educate patients appropriately. A failure to educate patients with possible shigellosis about the importance of handwashing and avoiding activities such as swimming, food preparation, and sexual activity could result in the spread of infection in households and communities, especially since *Shigella* can be spread by only a few organisms [7]. Also, it is important that HCP diagnose shigellosis and appropriately treat those who meet treatment criteria, as failure to treat could result in more severe clinical outcomes for certain patient populations. Furthermore, HCP knowledge of *E. coli* and other foodborne bacterial pathogens has also been found to be poor, further highlighting an overall need for both provider and patient education surrounding causes, treatment, and prevention of acute diarrhea [21–23]. All patients with an acute diarrheal illness, regardless of pathogen, would benefit from comprehensive health education to prevent spread of infection in the home or community. Federal public health practitioners can support HCP by updating information sources HCPs use most often, including federal guidance, regarding *Shigella* as well as other enteric pathogens. An example is CDC’s shigellosis website, which describes the spectrum of transmission routes, populations at increased risk, and recent changes in resistance for *Shigella* [1]. State and local public health practitioners can also support HCP by creating and distributing public health guidance for HCP in their jurisdictions.

Emerging resistance to first-line drugs threatens available treatment options [14, 16, 35] and some at-risk groups, including MSM and international travelers, have an elevated risk of resistant *Shigella* infection [16, 25, 36]. Although one study found high concordance (90%) for antibiotic prescriptions with prescribing guidelines [37], given high rates of antibiotic resistance to oral treatment agents [15], HCP should consider whether treatment will be beneficial for patients with *Shigella*. If treatment is needed, requesting AST can help to tailor treatment appropriately once results are available. Public health practitioners can help guide clinical practice by providing updated information about drug-resistant *Shigella* and highlighting the importance of obtaining susceptibility testing data. CDC’s “National Antimicrobial Resistance Monitoring System (NARMS) Now: Human Data” platform provides updated antibiotic resistance surveillance data for many enteric pathogens at the national and state level and can be utilized by HCP when they are considering treatment options for patients with shigellosis [15].

This study is subject to several limitations. First, the cross-sectional survey design, which asks about one point in time, may not represent HCP practice over time and could be impacted by several factors, including recent patient encounters, recent shigellosis outbreaks, testing availability, survey fatigue, and disruptions to the healthcare system during the COVID-19 pandemic. Similarly, social desirability bias may have influenced the results, as the source of information was self-report; however, antibiotic use reported in this study was consistent with previously described prescribing practices [37]. The survey questions and instructions may also have been interpreted incorrectly by some providers, or none of the answer options might have fit actual practice, causing HCP to choose a limited number of responses or choosing a response that was not reflective of their practice. Additionally, some providers may have had different responses than others*,* as disease severity and geographic distribution varies among different species of *Shigella* and may impact testing and prescribing practices [4, 30]. Although provider specialty was assessed, it is possible that some of the participants might not treat patients with diarrheal diseases or shigellosis due to scope of practice, which could impact their response options. We attempted to address this limitation by restricting analyses to only those who reported seeing patients with acute diarrhea or shigellosis. Also, although an attempt was made to recruit a diverse panel of providers in terms of specialty, geographic location, and type of practice, this is not a representative sample, and the results cannot be generalized to all HCP. Finally, we were limited in the number of questions we were able to include in the survey and had to focus questions we thought were most important for assessing knowledge and characterizing clinical management for shigellosis.

## Conclusions

Healthcare providers are essential in the prevention and control of shigellosis in the United States as they diagnose, treat, and educate those with symptomatic *Shigella* infection. However, this study suggests that providers’ knowledge of shigellosis transmission, risk groups, and treatment considerations could be improved by focused educational initiatives. Specific gaps in knowledge were identified surrounding MSM populations and risk of transmission through person-to-person contact. HCP, especially those providing care to individuals at increased risk of shigellosis, can contribute to stopping the spread of this infection in vulnerable populations by improving their knowledge of shigellosis transmission, prevention, and treatment.

### Supplementary Information

**Additional file 1.**

## Data Availability

The data that support the findings of this study are available from Porter Novelli Public Services. Restrictions apply to the availability of these data, which were accessed under a licensing agreement.
