# Supplementary material for: Healthcare providers’ knowledge and clinical practice surrounding shigellosis — DocStyles Survey, 2020
Source: BMC Prim Care. 2023 Dec 13;24:267. doi: 10.1186/s12875-023-02213-3 (PMC10717126; doi:10.1186/s12875-023-02213-3)
Supplement: Supplementary file 1 — Additional file 1. [file 12875_2023_2213_MOESM1_ESM.docx]

Healthcare providers’ knowledge and clinical practice surrounding shigellosis — DocStyles Survey, 2020

**SUPPLEMENTARY MATERIAL**

**Supplementary Figure S1: Sources of information healthcare providers report using to inform antibiotic treatment for acute diarrhea and *Shigella* infection**

“Acute Diarrhea” category represents only respondents who report seeing at least one patient per week with acute diarrhea (N=1287). “Positive *Shigella* test” category represents only respondents who report seeing at least one patient with *Shigella* each month (N=391).

**Supplementary Table S1: Questions asked of survey participants regarding *Shigella* and acute diarrhea**

| **Question** | **Answer Choices** |
| --- | --- |
| 1. What are some ways adults get infected with *Shigella*? *Select all that apply.* | Contaminated Food  Contaminated Water  Touching fomites  Person-to-person contact  During International Travel  Sexual activity  None of these |
| 2. Who is at risk for *Shigella* infection?  *Select all that apply.* | Children  Refugees  International travelers  People living in poverty  Men who have sex with men  People experiencing homelessness  None of these |
| 3. Which behaviors do you encourage adult patients (18+) who are actively sick with *Shigella* infection to follow?  *Select all that apply.* | Avoid swimming  Avoid preparing food for others  Wash hands frequently  Avoid sex  None of these  Question not applicable to me |
| 4. What makes it hard to talk to your patients with a *Shigella* infection?  *Select all that apply.* | Lack of time  Don’t have available educational resources  Diagnosis occurs after having seen patient  Not needed  It is not hard to provide health education  Question not applicable to me |
| 5. Which group best describes your patient population? *Select one.* | Adults  Children  All ages  Travel clinic  Sexual health clinic  None of these |
| **6. Where do you currently provide a consult for most patients with acute diarrhea?** *Select all that apply.* | In person, with the patient at your practice location  Remotely, with the patient at another clinical location  Remotely, with the patient not at a clinical location  None of these |
| 7. On average, how many patients per week do you see with acute diarrhea? If none, please type in ‘0’. | [Number box] |
| 8. When you provide a consult for a patient with acute diarrhea, do you routinely: *Select all that apply.* | Ask about sexual practices  Provide sexual health education  Use a culture-independent diagnostic test  Request a stool culture  Request antibiotic susceptibility testing  Treat with antibiotics  None of these |
| 9. Which of the following types of patients with acute diarrhea do you usually treat empirically with antibiotics before a pathogen is identified?  *Select all that apply.* | Adults  Children  International travelers  Men who have sex with men  Hospitalized patients  None of these |
| **10. When you prescribe antibiotics for patients with acute diarrhea, which antibiotics do you commonly prescribe before a pathogen is identified?** *Select all that apply.* | Ampicillin or amoxicillin  Azithromycin  Ciprofloxacin or another fluoroquinolone  Third generation cephalosporin  Trimethoprim-sulfamethoxazole  None of these |
| 11. What informs your antibiotic treatment for patients with acute diarrhea? *Select all that apply.* | Scientific articles, textbooks, or professional organization guidelines  Recommendations and information from CDC  Guidelines or data from your institution  Antibiotic susceptibility test results for the patient  Personal preference or advice from colleagues  Patient request or preference  None of these |
| 12. On average, how many patients per month do you see with a positive test for *Shigella*? If none, please type in ‘0’. | [Number box] |
| 13. Among patients with a positive test for *Shigella,* to whom do you usually prescribe antibiotics? *Select all that apply.* | Adults  Children  International travelers  Men who have sex with men  Hospitalized patients  None of these |
| **14. If you were to prescribe antibiotics for patients** with a positive test for *Shigella*, w**hich antibiotics would you usually prescribe?** *Select all that apply.* | Ampicillin or amoxicillin  Azithromycin  Ciprofloxacin or another fluoroquinolone  Third generation cephalosporin  Trimethoprim-sulfamethoxazole  None of these |
| 15. What informs your antibiotic treatment of patients with a positive test for Shigella?  Select all that apply. | Scientific articles, textbooks, online training, or professional guidelines  Recommendations and information from CDC  Guidelines or data from your institution  Antibiotic susceptibility test results for the patient  Personal preference or advice from colleagues  Patient request or preference  None of these |

| **Supplementary Table S2: Demographic and clinical practice characteristics of providers who provided correct answers to knowledge-based questions^a^** | | | | | | | |
| --- | --- | --- | --- | --- | --- | --- | --- |
|  |  | **Question** | | | | |  |
|  | **Total Respondents in Category** | **What are some ways adults get infected with *Shigella*?**  **Respondents with all correct responses (%)^a^** | **Chi-square**  **p-value^b^** | **Who is at risk for *Shigella* infection?**  **Respondents with all correct responses (%)^a^** | **Chi-square p-value^b^** | **Which behaviors do you encourage adult patients (18+) who are actively sick with *Shigella* infection to follow?**  **Respondents with all correct responses (%)^a, c^** | **Chi square p-value^b^** |
| **Total** | 1503 | 106 (7.1) |  | 316 (21.0) |  | 330 (24.2) |  |
| **Gender** |  |  | 0.51 |  | 0.10 |  | 0.75 |
| Male | 905 | 67 (7.4) |  | 203 (22.4) |  | 201 (23.9) |  |
| Female | 598 | 39 (6.5) |  | 113 (18.9) |  | 129 (24.6) |  |
| **Age** |  |  | **<0.01** |  | **0.04** |  | **0.03** |
| 25-34 years | 216 | 7 (3.2) |  | 35 (16.2) |  | 44 (22.3) |  |
| 35-44 years | 471 | 22 (4.7) |  | 91 (19.3) |  | 87 (20.3) |  |
| 45-54 years | 438 | 37 (8.4) |  | 94 (21.5) |  | 104 (26.2) |  |
| 55-64 years | 282 | 33 (11.7) |  | 76 (27.0) |  | 79 (30.3) |  |
| >65 years | 96 | 7 (7.3) |  | 20 (20.8) |  | 16 (19.3) |  |
| **Specialty** |  |  | 0.63 |  | 0.41 |  | 0.10 |
| Family Practitioner | 441 | 32 (7.3) |  | 89 (20.2) |  | 84 (20.2) |  |
| Internist | 559 | 37 (6.6) |  | 127 (22.7) |  | 127 (24.1) |  |
| Pediatrician | 252 | 20 (7.9) |  | 57 (22.6) |  | 57 (27.4) |  |
| Nurse Practitioner | 134 | 12 (9.0) |  | 24 (17.9) |  | 35 (31.0) |  |
| Physician Assistant | 117 | 5 (4.3) |  | 19 (16.2) |  | 27 (26.2) |  |
| **Region** |  |  | 0.04 |  | 0.11 |  | **0.01** |
| Midwest | 338 | 31 (9.2) |  | 73 (21.6) |  | 91 (29.2) |  |
| South | 329 | 14 (4.3) |  | 67 (20.4) |  | 63 (20.9) |  |
| Northeast | 513 | 32 (6.2) |  | 94 (18.3) |  | 96 (20.8) |  |
| West | 323 | 29 (9.0) |  | 82 (25.4) |  | 80 (27.6) |  |
| **Community Setting** |  |  | 0.40 |  | 0.75 |  | 0.70 |
| Urban | 543 | 37 (6.8) |  | 113 (20.8) |  | 118 (23.6) |  |
| Suburban | 777 | 60 (7.7) |  | 168 (21.6) |  | 176 (25.0) |  |
| Rural | 183 | 9 (4.9) |  | 35 (19.1) |  | 36 (22.2) |  |
| **Work Setting** |  |  | 0.23 |  | **0.03** |  | 0.34 |
| Individual Outpatient practice | 247 | 22 (8.9) |  | 52 (21.1) |  | 61 (27.9) |  |
| Group Outpatient practice | 1047 | 74 (7.1) |  | 234 (22.3) |  | 225 (23.7) |  |
| Inpatient Practice | 209 | 10 (4.8) |  | 30 (14.4) |  | 44 (22.1) |  |
| **Years in Practice** |  |  | **<0.01** |  | 0.52 |  | 0.38 |
| <10 years | 435 | 15 (3.4) |  | 82 (18.9) |  | 83 (21.0) |  |
| 10-19 years | 551 | 47 (8.5) |  | 116 (21.1) |  | 127 (25.2) |  |
| 20-29 years | 367 | 32 (8.7) |  | 83 (22.6) |  | 86 (26.0) |  |
| >30 years | 150 | 12 (8.0) |  | 35 (23.3) |  | 34 (25.2) |  |
| **Patients Seen per week** |  |  | 0.17 |  | 0.09 |  | 0.37 |
| <50 | 129 | 4 (3.1) |  | 22 (17.1) |  | 23 (20.4) |  |
| 50-99 | 580 | 41 (7.1) |  | 109 (18.8) |  | 127 (24.8) |  |
| 100-149 | 561 | 47 (8.4) |  | 136 (24.2) |  | 134 (25.8) |  |
| >150 | 233 | 14 (6.0) |  | 49 (21.0) |  | 46 (20.8) |  |
| **Pediatric Patients seen** |  |  | 0.98 |  | 0.99 |  | 0.23 |
| Yes | 1065 | 75 (7.0) |  | 224 (21.0) |  | 223 (23.3) |  |
| No | 438 | 31 (7.1) |  | 92 (21.0) |  | 107 (26.3) |  |
| Boldface indicates statistical significance (*p*<0.05).  ^a^ Percentages represent respondents who selected all correct responses for knowledge-based questions (Questions 1–3). Correct answers included all answer choices except “None of these”.  ^b^ Chi-squared p-values reflect the comparison between characteristics of HCP who answered all questions correctly  ^c^ Excludes 137 respondents who selected “Question not applicable to me”. (N=1366) | | | | | | | |
